# In silico prediction, characterization, docking studies and molecular dynamics simulation of human p97 in complex with p37 cofactor

**DOI:** 10.1186/s12860-022-00437-2

**Published:** 2022-09-10

**Authors:** Abolfazl Mirzadeh, George Kobakhidze, Rémi Vuillemot, Slavica Jonic, Isabelle Rouiller

**Affiliations:** 1 Department of Biochemistry and Pharmacology, Bio21 Molecular Science and Biotechnology Institute, The University of Melbourne, Parkville, VIC Australia; 2 IMPMC - UMR 7590, CNRS, Sorbonne Université, Muséum National d’Histoire Naturelle, Paris, France

**Keywords:** p97, p37, Homology modelling, Molecular docking, Molecular dynamics simulation

## Abstract

**Background:**

The AAA + ATPase p97 is an essential unfoldase/segragase involved in a multitude of cellular processes. It functions as a molecular machine critical for protein homeostasis, homotypic membrane fusion events and organelle biogenesis during mitosis in which it acts in concert with cofactors p47 and p37. Cofactors assist p97 in extracting and unfolding protein substrates through ATP hydrolysis. In contrast to other p97ʼs cofactors, p37 uniquely increases the ATPase activity of p97. Disease-causing mutations in p97, including mutations that cause neurodegenerative diseases, increase cofactor association with its N-domain, ATPase activity and improper substrate processing. Upregulation of p97 has also been observed in various cancers. This study aims towards the characterization of the protein–protein interaction between p97 and p37 at the atomic level. We defined the interacting residues in p97 and p37. The knowledge will facilitate the design of unique small molecules inhibiting this interaction with insights into cancer therapy and drug design.

**Results:**

The homology model of human p37 UBX domain was built from the X-ray crystal structure of p47 C-terminus from rat (PDB code:1S3S, G) as a template and assessed by model validation analysis. According to the HDOCK, HAWKDOCK, MM-GBSA binding free energy calculations and Arpeggio, we found that there are several hydrophobic and two hydrogen-bonding interactions between p37 UBX and p97 N-D1 domain. Residues of p37 UBX predicted to be involved in the interactions with p97 N-D1 domain interface are highly conserved among UBX cofactors.

**Conclusion:**

This study provides a reliable structural insight into the p37-p97 complex binding sites at the atomic level though molecular docking coupled with molecular dynamics simulation. This can guide the rational design of small molecule drugs for inhibiting mutant p97 activity.

## Introduction

Homotypic membrane fusion events occur to reassemble organelles such as the Golgi apparatus and the endoplasmic reticulum (ER) after mitosis [1]. One essential factor of this organelle biogenesis has been identified to be the AAA + (ATPases associated with diverse functions) ATPase enzyme p97 (Cdc48 in yeast). p97 is implicated in many cellular functions such as protein homeostasis, genome stability, autophagy pathways and membrane fusion [2]. The p97 monomer consists of an N-terminal domain and two conserved AAA ATPase (D1 and D2). The biological assembly of p97 forms a homohexamer with two concentric rings in which each monomer consists of an N-terminal domain and two conserved AAA ATPase (D1 and D2) domains [3]. The N-domain is the most dynamic region of the protein, shifting between an upwards or downwards (coplanar) conformation based on nucleotide state [4]. This domain can be further divided into two subdomains: Nn and Nc, composed of a double Ψ-barrel and a β-barrel respectively [5]. The major role of the N-domain is to recruit cofactors and adaptor proteins, many of which bind to the hydrophobic cleft between Nn and Nc [6]. The activity of p97 is modulated by a multitude of cofactors and often dependent on ubiquitination [7]. p97 in complex with specific cofactors allows this enzyme to perform its unfoldase/segragase activity against a variety of protein substrates and in different cellular processes.

Mutations in p97 are associated with neurodegenerative conditions collectively referred to as multisystem proteinopathy (MSP1) [8–10]. These disease mutants possess increased ATPase activity and modify higher affinity for cofactors which can lead to improper degradation of substrates. The reliance on p97’s functions results in upregulated expression in some cancers due to the heightened risk of proteotoxic stress [11]. The design of small molecules that interfere with the binding and assembly of cofactors is an attractive alternative to ones that directly and uniquely bind to p97. However, the lack of complete high-resolution structures of p97-cofactor complexes impairs development of cofactor-specific inhibitors.

The interaction between p97 and its cofactors p37 and p47 have been shown to be required for organelle biogenesis. p47 is required for the reassembly of Golgi fragments and for ER network formation [12–14]. This includes a mechanism requiring interaction with the deubiquitinase VCIP135 [15], and the t-SNARE syntaxin 5 [16]. p37 is similarly essential for Golgi and ER reassembly in interphase and telophase, interacting instead with the SNARE GS15 [17]. p47 regulates p97 by overall reducing ATPase activity [18], while p37 increases it [19]. Due to this role, p37 has been identified as the first known activating cofactor of p97. In addition, it has been shown that in p97 disease mutants, both p37 and p47 cofactors lose their ability to modify the ATPase activity of the D2 domain [19]. This suggests that the cellular functions of p37 and p47 could be impaired when associated with mutant p97. However, the effect of p97 disease mutants on homotypic membrane fusion events or organelle biogenesis has not yet been observed.

Both p37 and p47 share similar domains and motifs. They both have a C-terminal UBX (ubiquitin regulatory X) domain which allows binding to the p97 N-terminal [20]. They also have a SEP (domain and a SHP motif, the latter of which proposed to binds to an alternative site in the p97 N-domain [21]. The major difference between p37 and p47 is that p37 lacks an N-terminal UBA (ubiquitin-associating) domain. This domain is required for recognition and binding of monoubiquitin in p47 [22]. Furthermore, amino acids 69–92 in p47 have been shown to be responsible for its inhibition of ATPase activity. It has been shown that in the absence of this region, p47 turns into activating cofactor like p37 [19].

UBX domain-containing cofactors bind to p97 through docking the UBX domain to the hydrophobic groove between Nn and Nc [6]. A high-resolution structure of the p47 UBX domain bound to the p97 N-domain (PDB code: 1S3S) revealed that a conserved S3/S4 loop in UBX binds to the hydrophobic groove [23]. More recently, cyro-EM structures of full-length p47 bound to a R155H p97 mutant (PDB codes: 7R7S, 7R7T) revealed a mechanism of dysregulation of cofactor binding and interprotomer communication in p97 mutants [24]. The structures of UBA (PDB code: 1V92 [25]), SEP (PDB codes: 1VAZ, 1SS6 [25, 26]) and UBX (PDB codes: 1JRU, 1I42 [25]) domains of p47 have been determined separately.

Currently, there is no structural characterisation of p37 or any of its domains. As previously described, p37 is involved in alternative pathways for membrane fusion and organelle biogenesis. Structural analysis of p97-p37 binding is necessary to understand its mechanism in regulating p97 activity and role in these cellular processes. To discover insights into the mechanism of p97-p37 binding, this study utilised homology modelling to predict structures of p37 UBX domain and protein–protein docking to simulate the binding of the p37 UBX to the p97 N-D1domain.

Homology modelling software utilises proteins with previously determined structures to predict proteins of unknown function. Servers such as PHYRE2 and I-TASSER were used to predict the structure of the p37 UBX domain [27–29].

In this project, HDOCK and HAWKDOCK were applied as a prediction docking server to characterising the binding of p37 UBX and p97 N-D1 domain. Furthermore, the key residues for PPIs are highlighted by the MM/GBSA program based on the binding free energy (kcal/mol) [30]. The mCSM server and multiple sequence alignment were applied to validate the selected key residues involved in the binding site and their conservatively among other UBX cofactors. The mCSM server is used to analyse the impact of a mutation in the binding site to determine the relative contribution of each residue in stabilising the protein–protein interaction [31]. MD produces a more accurate biophysical simulation of protein–protein binding, overcoming the limitation of rigid docking approaches in HAWKDOCK and HDOCK. Therefore, after molecular docking, we performed molecular dynamics (MD) simulation to assess the validity of the binding pose prediction [32]. MD is an essential tool for simulating the biological motions of the proteins. The MD simulation explores the conformational space and predicts the changes of interactions of the targeted complex. MD simulations are widely used to validate the prediction of molecular docking [21, 33, 34]. In such cases, the absence of major conformational changes during a sufficiently long MD simulation is good evidence of the stability of the predicted docking pose. It also provides good information about binding interaction such as hydrogen bonding.

## Methods

### Secondary and tertiary structure prediction of p97 cofactors

Homology modelling was performed to predict the secondary and tertiary structure of the human p37 cofactor (Uniprot ID: Q14CS0) using SWISS-MODEL, PHYRE 2 and I-TASSER servers [27, 28, 35]. The result obtained from each server was compared with the experimentally determined structure of the template to validate the predicted secondary and tertiary structures of p37.

### Model validation of predicted 3D model

Model Validation was applied to validate the accuracy of predicted tertiary structures and detect the most accurate candidate model among them. The predicted models were assessed by the quality assessment tools Qualitative Model Energy Analysis (QMEAN) [36], and the Ramachandran plot.

### Molecular docking between p97 and p37

HDOCK and HAWKDOCK were applied to conduct molecular docking based on blind docking for detection of possible binding sites between UBX domain of human p37(Uniprot ID: Q14CS0) and N-D1 domains of human p97(Uniprot ID: P55072) to find favourable protein–protein complex pose. [30, 37]. At the first step, HDOCK was used to find the best pose of the modelled complex based on a template. The predicted model of the p37 UBX domain as a ligand was docked with the p97 N-D1 from human as a receptor. In the next step, the PDB code of the top ranked docked model was analysed by PyMOL version 2.3.4 [38] and Arpeggio [39] to calculate the distance between atoms (Cα) of the interacting residues and determine the type of interaction. These were used to inform restraints which were then applied to protein docking performed by HAWKDOCK. Finally, the mCSM server was used to evaluate the impact of mutations of p37 UBX interacting residues on binding affinity between p37 UBX and p97 N-D1 domain as well as the stability of the protein complex [31]. In addition, Geneious Prime (version 2021.1.1) was used to perform multiple sequence alignment for determination of residues of the UBX domain among different cofactors that interact with the p97 N-D1 domain.

### Molecular dynamics simulation for p37 UBX domain—p97 N-D1 domains complex

The top docked model of the p97 N-D1 domain in complex with p37 UBX domain obtained from molecular docking and the MM/GBSA program was further analyzed using molecular dynamics simulation to ensure the conformational stability of the p97 N-D1—p37 UBX complex in a solvated model system over time of simulation following methodology described by others [32, 40, 41]. The MD simulation was performed for 100 ns starting from the predicted docking pose between p97 N-D1 domain and p37 UBX domain. The complex was solvated with TIP3 water molecules in Periodic Boundary Conditions (PBC) with margin of 15 Å. Na + and Cl- ions were added to neutralize the total charge of the system. CHARMM36 [42, 43] was used to model the system. Energy minimization was performed with positional restraint on the backbone of the proteins. After the energy minimization, the system was heated up from 0.1 K to 300 K during 0.1 ns of MD simulation, by slowly increasing the temperature by 3 K each picosecond. The positional restraint on the backbone is kept preserving the structure during the heating. The system was equilibrated during another 0.1 ns simulation without positional restraint. The production run was performed during 100 ns of simulation with a timestep of 2 femtoseconds. The temperature and pressure were controlled by Langevin thermostat at 300 K and Langevin barostat at 1 atm. The long-range electrostatic interactions were calculated by Particle Mesh Ewald (PME) method and the Lennard–Jones potential was switched off at 10 Å. MD simulations were carried out using GENESIS version 1.6.1. [44]

## Results

### Prediction of secondary structure of human p37

The secondary structure of human p37 (Uniprot ID: Q14CS0) was predicted using homology modelling via three different prediction servers including PHYRE2, SWISS-MODEL and I-TASSER. The N-terminal domain of p37 from residues 1 to 136 was predicted as a disordered region. The SEP domain of human p47 (Uniprot ID: Q9UNZ2, PDB code: 1SS6) and the C terminus from rat p47 (Uniprot ID: O35987, PDB code: 1S3S) were chosen as templates for the SEP and UBX domain of human p37, respectively. Although many regions of p37 obtained from these servers were similar, the secondary structure of p37 predicted by PHYRE2 was more consistent with those of templates (data not shown). According to the PHYRE2 prediction server, the p37 SEP domain is comprised of two ⍺-helices (166–176, 182–187) and three β-strands (142–150, 153–154, 193–199). Moreover, the p37 UBX domain is composed of two ⍺-helices (279–289, 317–319) and four β-strands (256–269, 268–273, 299–301, 324–329).

### Prediction of tertiary structure of human p37

**Fig. 1:**
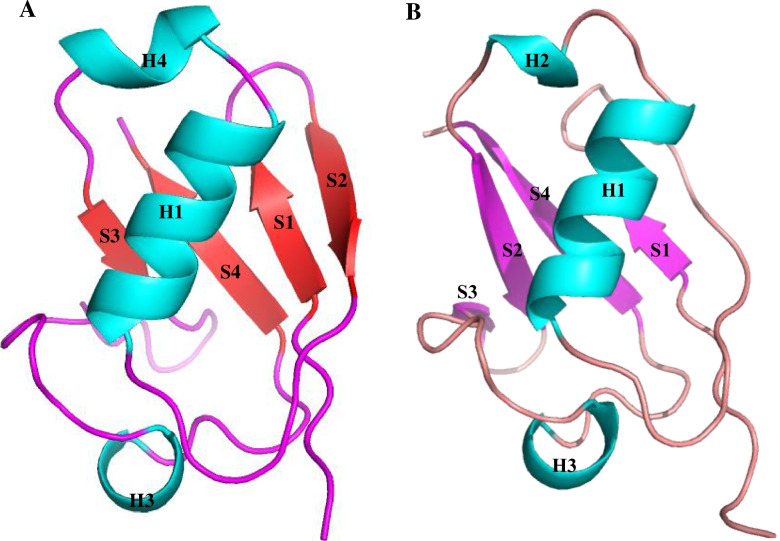
**A** Sequence alignment between UBX domain of p37(Uniprot ID: Q14CS0) and C-terminus from rat (PDB code: 1S3S) of p47(Uniprot ID: O35987). **B** Predicted tertiary structure of p37 UBX by PHYRE2 server(left), predicted tertiary structure of p37 UBX by I-TASSER server(right)

Since p37 binds to the p97 N domain through the UBX domain [17, 45], the tertiary structure of this domain was predicted by homology modelling via SWISS-MODEL, PHYRE2 and I-TASSER. The selected template for the p37 UBX domain was the C-terminus of p47 from rat (Uniprot ID: O35987, PDB code: 1S3S), indicating that the UBX domain is highly conserved between p37 and p47. According to the predicted tertiary structure by both SWISS-MODEL and PHYRE2, 77 residues from residue 252 to 329 belonging to the human p37 UBX domain have been modelled based on the C-terminus of p47 from rat, with 100.0% confidence and 64% identity (Fig. 1A). Meanwhile, The UBX domain of p37 was predicted by I-TASSER in which the final model was generated based on the top 10 threading templates (Fig. 1B). Therefore, the predicted models were further verified using different validation methods to detect the more accurate model.

### Model validation by different methods

QMEAN server is a composite scoring function that estimates global (entire structure) and local (residue) quality of the predicted model [46]. The quality of the predicted model is valued between 0 and 1, and each model has more reliability when the score is closer to 1. According to the QMEAN, the score of the 3D model predicted by PHYRE2 was 0.72. Meanwhile, the QMEAN score of predicted models obtained from I-TASSER was 0.61. This result indicates that although both predicted models are of good quality, the model predicted by PHYRE2 is more optimal due to higher quality.

**Fig. 2:**
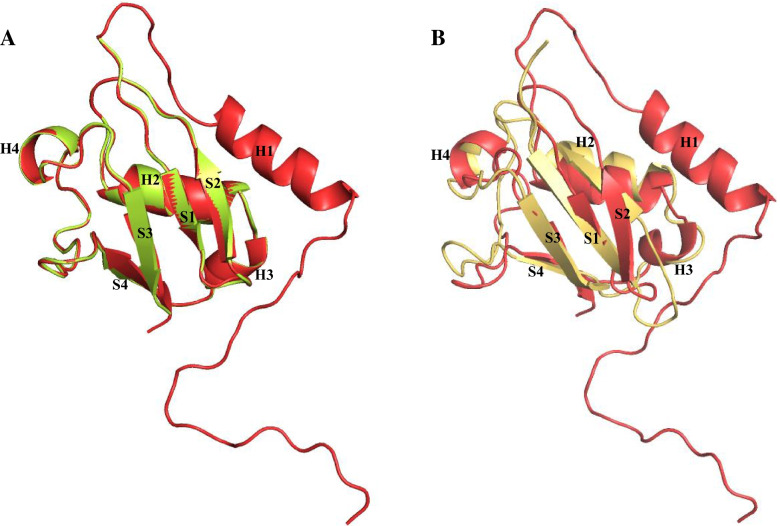
Overlay of template and predicted models. **A** Superpositions of p37 UBX (yellow) predicted by PHYRE2 server with p47 UBX solution structure (PDB code: 1S3S, G). **B** Superpositions of p37 UBX (yellow) predicted by I-TASSER server with p47 UBX template

In addition, the overall root mean square deviation (RMSD) score for the predicted p37 UBX domain via PHYRE 2 and I-TASSER based on the template (1S3S) were 0.51 and 3.42, respectively (Fig. 2).

Moreover, according to the Ramachandran analysis, 87.8% of residues in the p37 UBX model predicted by PHYRE2 are within the favored region, while only 81.8% of residues in the model predicted by I-TASSER are in the favored region (Table 1). Therefore, the 3D model predicted by PHYRE2 is more accurate than that of I-TASSER, as a greater number of residues are in the favored region.

**Table 1:** Validation of 3D model predicted by different servers by Ramachandran plot

UBX domain predicted by different servers	Number of residues in favored region	Number of residues in allowed region	Number of residues in outlier region
p37 UBX Predicted by PHYRE2	65 (87.8%)	3 (4.1%)	6 (8.1%)
p37 UBX Predicted by I-TASSER	63 (81.8%)	10 (13.0%)	4 (5.2%)

### Protein docking between p97 and p37 UBX domain

**Fig. 3:**
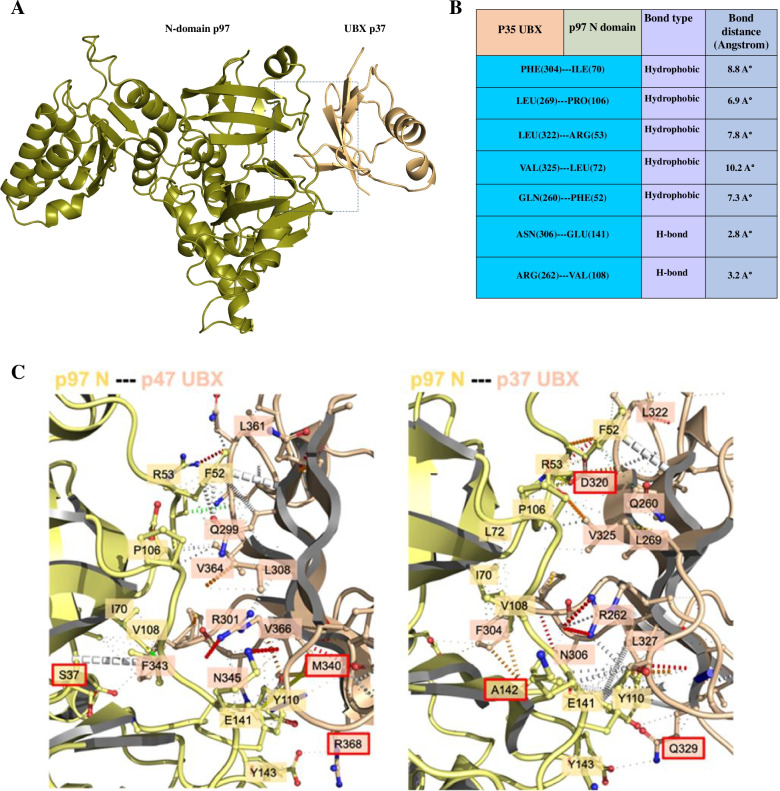
The best docked pose obtained by HDOCK and HAWKDOCK. **A** Top docked model complex between p37 UBX from human and p97 N-D1 from human. **B** The key residues involved in interaction site between p37 UBX and p97 N domain. **C** Interatomic interactions between p97 N-domain bound to the UBX domain of either p47 (left) or p37 (right) calculated by Arpeggio. The p97-p47 complex was obtained from PDB code: 1S3S, and the p97-p37 complex is from the best docked model obtained by HDOCK and HAWKDOCK. Small grey dashed lines indicate hydrophobic interactions. White dashed lines indicate π-stacking interactions. Blue and thick white dashed lines indicate aromatic-amide interactions. Red and orange dashed lines indicate polar interactions. Residues highlighted in red indicate that the interaction or residue itself is not conserved across cofactors

**Fig. 4:**
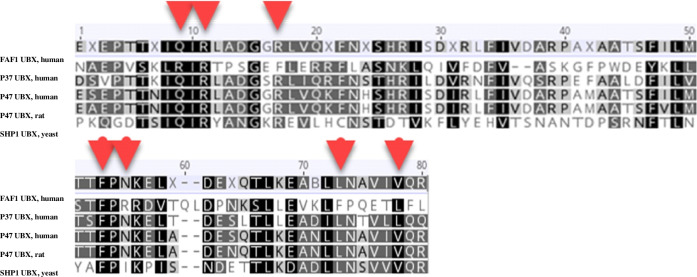
Multiple sequence alignment of UBX domain among different cofactors

According to the HDOCK server, the best docked pose for p37 UBX in complex with p97 N-D1, was similar to the X-ray solved structure of p97 in complex with p47-UBX from rat (PDB code: 1S3S; RMSD score of 0.56 Å). After calculating the distance between carbon α of the interacting residues, HAWKDOCK server was used to predict the best matching binding mode of p37 UBX to p97 N-D1 domain based on different restraints. Finally, the best docked pose was detected according to the HAWKDOCK score (-3647.93) and MM/GBSA, a functionality of HawkDock sever, with the free binding energy of -41.21 kcal/mol (Fig. 3). The key residues with the lowest free binding energy involved in the interaction site between p37 UBX and p97 N-D1 domain were determined by MM/GBSA (Table 2). Intriguingly, all selected key residues with the lowest binding free energy were consistent with the key residues engaged in the interaction site between p47 UBX domain and p97 N-D1 domain. Meanwhile, the position of interacting conserved residues was specified within the 3D model of p37 UBX. The result showed that GLN(260) is located in beta-strand 1, ARG(262) is located in the turn between Strand1 and 2. LEU(269) is located in strand 2. PHE(304)& ASN(306) are located in the turn between strand4 and helix 4, and LEU(322) and VAL(325) are located in strand 5. Intriguingly, these regions are conserved between the UBX domains of p37 and p47. In addition, according to the multiple sequence alignment, a large number of the residues involved in the binding interfaces are conserved among the UBX domain of different cofactors especially for p47 and p37 (Fig. 4) (Table 3).

**Table 2:** The interacting residues between p97 and p37 determined by MM/GBSA

Key residues of p37 UBX domain		Key residues of p97 N domain	
**Residue**	**Binding Free Energy (kcal/mol)**	**Residue**	**Binding Free Energy (kcal/mol)**
PHE(304)	-5.77	PHE(52)	-7.46
LEU(269)	-4.52	TYR(110)	-4.21
LEU(322)	-3.45	ARG(53)	-3.17
VAL(325)	-3.74	PRO(106)	-2.86
GLN(260)	-2.18	TYR(143)	-2.72
ASN(306)	-1.41	ARG(262)	-2.13
ARG(262)	-1.28	ILE(70)	-1.48

**Table 3:**
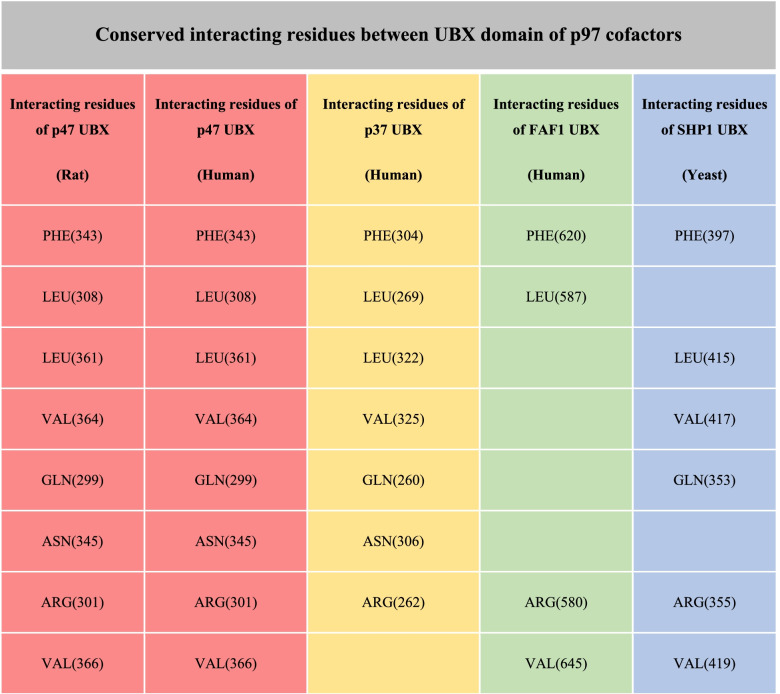
The conserved interacting residues among cofactors containing UBX domain. The number of each interacting residue of various cofactors is based on their sequence

### Predicting effects of mutations on stability of docked complex model

Mutation of selected interacting residues in the p37 UBX domain to ALA resulted in reduced binding affinity between p37 and p97, indicating that these conserved residues among UBX cofactors are essential in stabilising the interaction with the p97 N domain (Table 4).

**Table 4:** The impact of mutation of interacting residues on p37-p97 binding affinity

The effect of mutation on binding affinity		
**Interacting residues of p37 UBX domain**	**Predicted affinity change (ΔΔG affinity)**	**Binding affinity**
PHE(304)-ALA	-0.732 kcal/mol	Decreasing affinity
LEU(269)-ALA	-0.267 kcal/mol	Decreasing affinity
ASN(306)-ALA	-0.446 kcal/mol	Decreasing affinity
LEU(322)-ALA	-0.683 kcal/mol	Decreasing affinity
GLN(260)-ALA	-0.897 kcal/mol	Decreasing affinity
VAL(325)-ALA	-0.582 kcal/mol	Decreasing affinity
ARG(262)-ALA	-1.147 kcal/mol	Decreasing affinity

### RMSD and RMSF analysis of the MD simulation

**Fig. 5:**
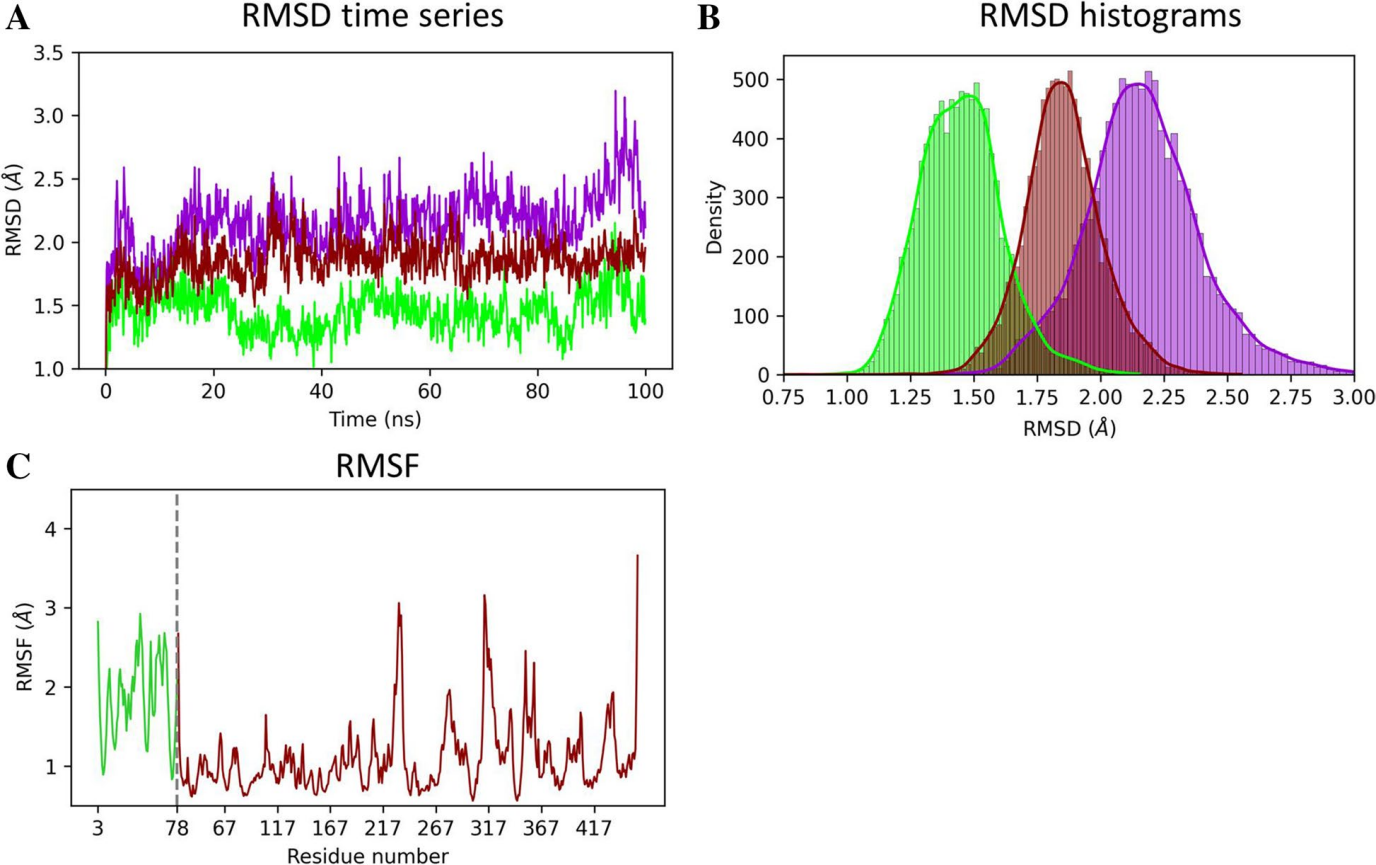
RMSD and RMSF analysis of the MD trajectory **A** RMSD as a function of the simulation time for p37 UBX domain (green), p97 N-D1 (red) and the complex p37 UBX—p97 N-D1 (purple). **B** Histograms of the RMSD for p37 UBX domain, p97 N-D1 and the complex p37 UBX—p97 N-D1. C) RMSF over the simulation of each individual residue for p37 UBX domain and p97 N-D1

To analyse the stability of the complex over the MD simulation, we calculated the root mean square deviation (RMSD) of each trajectory frame to the initial conformation. Time series and histograms of the RMSD are presented in Fig. 5A and Fig. 5B respectively. According to Fig. 5A, the RMSD of the complex is equilibrated at 5 ns and remain stable around 2.2 Å until the end of the simulation. The same behaviour is observed when considering the RMSD time series of p37 UBX and p97 N-D1 individually, with RMSD value stabilizing around 1.4 Å and 1.8 Å respectively, indicating a stable conformation all over the simulation. Observations of the histograms of the RMSD (Fig. 5B) show a distribution around a single peak for p37 UBX, p97 N-D1 and the p37 UBX-p97 N-D1 complex, suggesting that the explored conformations are all in the vicinity of the initial conformation. Considering the molecular size of p97 N-D1 and p37 UBX (approximatively, 70 Å and 30 Å respectively), the RMSD values obtained represent minor conformational changes. To evaluate the variation of the molecular structure during the MD simulation, we calculated the root mean square fluctuation (RMSF) over each residue of p37 and p97. The RMSF presented in Fig. 5C indicates a majority of residue with low fluctuation (around 1 Å) and peak values corresponding to loop regions. Besides, we observed the obtained MD trajectory visually using VMD. The visual checking confirms the presence of a single conformation explored during the MD simulation. According to the RMSD analysis, the RMSF analysis and the visual observation, the molecular structure of the complex remains stable for the duration of the MD simulation.

### Analysis of the stability of H-bond interactions between p37 UBX and p97 N-D1

**Fig. 6:**
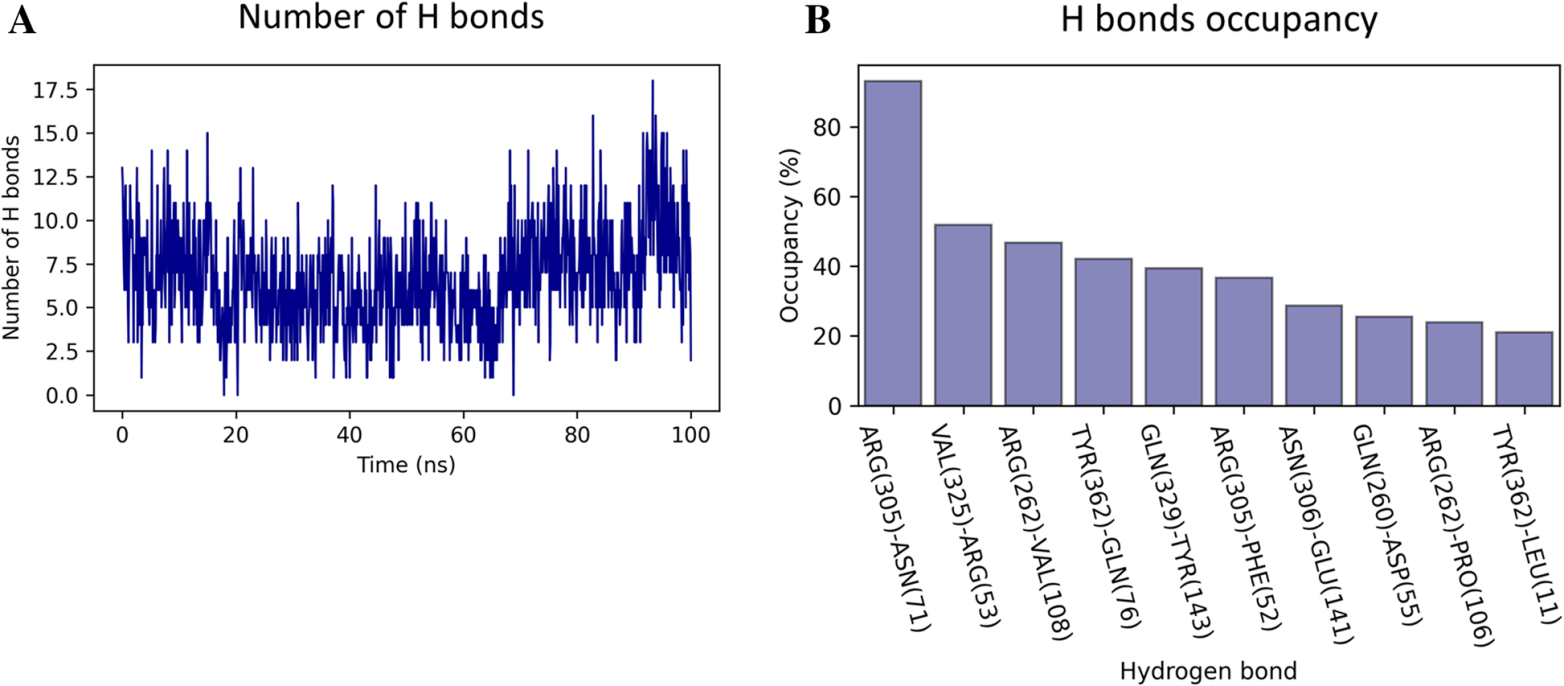
H-bond analysis between p37 UBX and p97 N-D1 **A** The number of H-bonds between p37 UBX and p97 N-D1 as a function of simulation time. **B** Occupancy of each H-bonds that are higher than 20% during the length of the simulation. The labels show the residue name and number of p37 UBX (first) and p97 N-D1 (second)

To ensure that the binding interaction between p37 UBX and p97 N-D1 is stable throughout the MD simulation, we counted the number of H-bonds formed between p37 UBX and p97 N-D1 at each step of the simulation. Figure 6A shows the number of H-bonds present as a function of the simulation time and Fig. 6B the H-bonds occupancy (the percentage of presence of each H-bond during the simulation). According to Fig. 6A, the number of H-bond remains stable during the first 70 ns of simulation around five H-bonds, then undergoes a small increase around seven H-bonds until the end of the simulation. The existence of a at least 2 H-bond interactions during the entire simulation suggests a strong stability of the binding between p37 UBX and p97 N-D1. Moreover, Fig. 6B shows that the H-bonds interactions estimated by the molecular binding (Fig. 3B), ARG (262)-VAL (108) and ASN(306)-GLU(141) remains among the H-bonds with the highest occupancy (47% and 36% respectively), suggesting that these H-bonds play a significant role in the binding between p37 UBX and p97 N-D1.

## Discussion

In this work, we studied the protein–protein interaction between p97 and the p37 cofactor via computational methods, which is essential for understanding the assembly of p97 and p37 in processes such as membrane fusion and organelle biogenesis. Since there is no structural information regarding p37 and its interaction with p97, this study aimed to provide the first structural insights prior to experimental methods, which often involve lengthy optimisation and analysis. Like p47, this cofactor is involved in Golgi and ER biogenesis and plays a pivotal role in their maintenance during interphase and their reassembly at the end of mitosis [17].

p37 is composed of two domains including SEP and UBX which have high similarity to those of p47. We characterized the secondary structure of p37 via homology modelling to determine the secondary structure features of the SEP and UBX domains. p37 belongs to the largest family of p97 cofactors named UBX proteins that are involved in the interaction with p97 N domain through their UBX domain. In addition, this domain contains interacting residues that are conserved among members of this family. Therefore, the tertiary structure of the p37 UBX domain was predicted based on homology modelling via three powerful and prevalent predication servers. Model validation analysis showed that the tertiary structure predicted by PHYRE2 was the most accurate. In this model, the p37 UBX domain was modelled based on the C-terminus of p47 from rat as a template, with 100.0% confidence and 64% identity.

Despite the fact that both p47 and p37 participate in Golgi and ER biogenesis and need VCIP135 for their function, there are some sharp differences in their structures and roles. The main structural difference between p37 and p47 is the lack of the UBA domain in the N-terminus of p37, which in p47 binds to monoubiquitinated substrates in the presence of p97. The amino acid region 69–92 found in p47 is also absent in p37 [22, 47]. Moreover, a biochemical binding assay showed that p37 does not bind to ubiquitin in either the presence or absence of p97. In addition, unlike p97/p47 complex, adding a ubiquitin mutant had no effect on p97/p37-dependent Golgi reassembly in vitro [17]. The other major difference is that although p47 inhibits the ATPase activity of p97, p37 increases it [47]. The mechanisms of regulation are not yet understood and requires a comparison between the structure of the p97-p47 and p97-p37 complexes. Therefore, despite the conserved residues in the UBX domains of these cofactors, it might seem that there are some different interacting residues within p47 that alter the conformation and activity of p97.

As there is no solved structure of p37 in complex with p97, we applied protein docking to model the interaction between p37 UBX and p97 N-D1 at the atomic level and characterize their interacting key residues to elucidate fundamental biochemical processes. The docking is based on two steps including the prediction of the ligand conformation, position, and orientation in terms of its receptor (referred to as *pose*) as well as assessment of the binding affinity [48]. These two steps are derived from sampling methods and scoring schemes, respectively. In addition, the molecular docking becomes more efficient and realistic if there is information about the experimental solved structure, which has the highest similarity to target model. This can help to find the best pose for docking and highlight the interacting residues of proteins engaged in the complex. Therefore, at the first step, HDOCK was used to find the best pose of model complex based on found template which has the highest identity with the query. The predicted model of p37 UBX domain as ligand was docked with the p97 N-D1 from human as a receptor. HDOCK opted the solved structure of p97 N-D1 in complex with p47 UBX domain from rat (PDB code: IS3S) as the best docked pose for p37 UBX complexed with p97 N-D1 with an RMSD score of 0.56 Å. In the next step, we used Arpeggio and PyMOL to visualise the interatomic interactions between the ligand and receptor and then measure the distance between atoms (Cα) of the interacting residues to determine restraints for further docking. Arpeggio is a powerful tool to calculate, visualize, and understand different types of interactions including van der Waals, ionic, carbonyl, hydrophobic, and hydrogen bonds for non-experimentally determined structures such as homology models or docking poses [49].

The candidate pose with the minimum energy value and top score was detected based on an affinity scoring function and binding free energy (kcal/mol) calculated by the HAWKDOCK algorithm score and the MM/GBSA program, respectively (Fig. 3). According to protein docking, it was found that there are several hydrophobic interactions between the p37 UBX and p97 N domain that might contribute to the stability of complex. The interacting residues in the interface of p37 UBX domain were GLN 260, Leu269, PHE 304, LEU 322, VAL 325 which participate in the binding sites with p97 N domain interacting residues PHE52, PRO106, ILE 70, ARG 53, LEU 72 and TYR 110. In addition, there are hydrogen-bonding interactions between p37 UBX residues ARG 262, ASN 306 and p97 N domain residues VAL108 and GLU 141, respectively.

Meanwhile, mCSM was applied to confirm that key residues involved in the interaction between p37 UBX and p97 N domain were essential to stabilising the complex. mCSM predicts the impact of mutations on protein–protein interactions and protein complex stability by determining free energy differences between wild-type and mutant residues [31]. We found that mutations of key residues reduced binding affinity with p97. Intriguingly, these interacting residues in the interface of the p37 UBX domain are conserved amongst those of other UBX cofactors especially with those of p47. Previous studies using surface plasmon resonance (SPR) determined binding affinities for p47-p97 and p37-p97 with K_D_ values of ∼20 nM and 29 nM, respectively [47]. Interestingly, this finding is consistent with our protein docking and multiple sequence alignment results regarding the role of three interacting residues in p47 UBX domain including THR 263, VAL 366, ARG 368 that are absent in p37, indicating the higher binding affinity of p47 towards p97 as compared to the latter.

The protein–protein interactions for complex having minimum binding energy (stronger binding) were further assessed by molecular dynamics simulations analysis. MD simulations studies were performed up to 100 ns through GENESIS to analyse dynamics and structure of complex. The analysis of RMSD and RMSF indicated the conformational stability of the predicted p97 N-D1 complexed with p37 UBX in the solvated model system throughout the simulation. Meanwhile, The H-bonds analysis confirmed a stable interaction between p37 UBX and p97 N-D1 during the simulation.

## Conclusion

This study provides the first structural insights into the p37-p97 complex based on homology modelling, protein–protein docking and molecular dynamics that identified key residues in the protein–protein interaction between p37 and p97. Our conclusion showed that how human p37 can interact with p97 and recruit it for the membrane fusion mechanism. Intriguingly, many interacting residues in p37 cofactor are conserved in p47, indicating that the significance of these conserved residues in interaction with p97. The results of our work on binding interactions between the p37 and p97 would be useful for subsequent studies, including understanding the mechanisms of membrane fusion events and identifying therapeutic targets for human disease within this system.

## Data Availability

All data generated or analysed during this study are included in this published article (and its supplementary information files).
